# Ru‐Doped Fe₂TiO₅ as a High‐Performance Electrocatalyst for Urea‐Assisted Water Splitting

**DOI:** 10.1002/smll.202412370

**Published:** 2025-03-10

**Authors:** Kassa Belay Ibrahim, Karim Harrath, Mohammadhossein Hamrang, Matteo Bordin, Stéphanie Bruyère, David Horwat, Enrique Rodríguez‐Castellón, Marshet Getaye Sendeku, Pratik Shinde, Danilo Oliveira de Souza, Luca Olivi, Alberto Vomiero, Elisa Moretti, Tofik Ahmed Shifa

**Affiliations:** ^1^ Department of Molecular Sciences and Nanosystems Ca’ Foscari University of Venice Via Torino 155 Venezia Mestre 30170 Italy; ^2^ GanJiang Innovation Academy Chinese Academy of Science Ganzhou 341000 P. R. China; ^3^ Université de Lorraine, CNRS, IJL Nancy F‐54000 France; ^4^ Departamento de Química Inorgánica Cristalografia y Mineralogía Facultad de Ciencias Instituto Interuniversitario de Investigación en Biorrefinerías I3B Universidad de Málaga Málaga 29071 Spain; ^5^ Ocean Hydrogen Energy R&D Center Research Institute of Tsinghua University in Shenzhen Shenzhen 518057 P. R. China; ^6^ Elettra‐Sincrotrone Trieste Strada Statale 14, km 163.5 Trieste 34149 Italy; ^7^ Division of Materials Science Department of Engineering Sciences and Mathematics Luleå University of Technology Luleå SE‐97187 Sweden

**Keywords:** Fe_2‐x_Ru_x_TiO_5_, Oxygen Evolution reaction, pseudobrookite, urea‐assisted water oxidation, Uria Oxidation reaction

## Abstract

The urea oxidation reaction (UOR), with its low thermodynamic potential, offers a promising alternative to the oxygen evolution reaction (OER) for efficient hydrogen production. However, its sluggish kinetics still demand the development of an efficient electrocatalyst. In this study, the critical role of Ru doping in Fe₂TiO₅ is demonstrated to accelerate UOR kinetics. The computational finding confirmed the feasibility of this approach, guiding the experimental synthesis of Fe_2−x_Ru_x_TiO_5_. Benefitting from surface properties and electronic structure, the synthesized material exhibits superior performance with a potential of 1.30 V at a current density of 10 mA cm^−2^ for UOR, compared to undoped Fe2TiO5 (1.40 V). Moreover, it demonstrates a favourable Tafel slope of 52 mV dec^−1^ and maintains robust durability for 72 h. As confirmed from experimental and computational findings, the enhanced activity can be attributed to the Ru doping resulting in structural distortion at the Fe site and creation of a favourable adsorption site thereby enhancing UOR via dual active center. This study not only broadens the potential applications of Fe2TiO5‐based materials beyond their traditional role as photocatalysts but also establishes them as promising electrocatalysts underscoring the versatility and improved performance of Fe_2−x_Ru_x_TiO_5_.

## Graphical abstract

Ru doping in Fe₂TiO₅ enhances urea oxidation reaction (UOR) kinetics by inducing structural distortion and creating favorable adsorption sites. The optimized _2−x_Ru_x_TiO_5_ exhibits superior performance with a lower onset potential and robust durability, demonstrating its potential as an efficient electrocatalyst beyond traditional photocatalytic application.

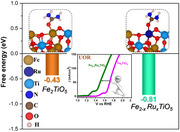

## 1 Introduction

Energy crisis and climate change are the two main challenges encountered recently and have created huge concerns worldwide. Research and development of new renewable energy sources that can replace conventional fossil fuels is of paramount importance.^[^ ^1^ ^]^ Renewable energy generation, that is, hydrogen fuel produced by seawater electrolysis^[^ ^2^ ^]^ through OER^[^ ^3^ ^]^ and HER has emerged as a viable option. Unfortunately, the kinetically sluggish nature of OER, the resulting high overpotential limit (1.23 V), and the high energy consumption required for efficient H_2_ production have significantly impeded the overall performance. Considering the overall water splitting, the OER process requires a high voltage to drive it; therefore, substituting OER with an electro‐oxidation reaction with a lower theoretical potential is an efficient methodology to alleviate this issue. Recently, as an alternative to traditional hydrogen production from electrocatalytic water splitting, many readily oxidizable molecules including alcohols,^[^ ^4^ ^]^ glucose,^[^ ^5^ ^]^ urea,^[^ ^6^, ^7^ ^]^ hydrazine,^[^ ^8^ ^]^ and 5‐(hydroxymethyl)furfural^[^ ^9^ ^]^ have been deployed to replace the OER process and improve energy efficiency. Owing to the favorable thermodynamics of UOR (CO(NH_2_)_2_ + 6OH^−^ → N_2_ + CO_2_ + 5H_2_O + 6e^−^ = 0.37 V) over the OER (1.23 V), this technology can theoretically realize ≈70% of energy saving compared with traditional electrocatalytic water splitting.^[^ ^10^, ^11^, ^12^, ^13^, ^14^ ^]^ Urea plays a significant role in enhancing water‐splitting processes, particularly through the UOR, which serves as an alternative to traditional methods for hydrogen production. This approach not only reduces energy consumption but also improves the overall efficiency of hydrogen generation. Therefore, UOR is a thermodynamically favorable process as compared to OER. Furthermore, urea can be considered a “valuable” waste. It is an environmental pollutant released by agricultural fertilizers, industrial processes, and domestic excretion, but it has a high hydrogen content (6.67 wt%).^[^ ^15^, ^16^, ^17^, ^18^ ^]^ Moreover, urea is universally available in human urine and industrial and sanitary wastewater, making its electrolysis highly feasible.^[^ ^19^, ^20^, ^21^, ^22^, ^23^ ^]^ However, the practical application of UOR is significantly hindered by major challenges associated with multistep electron transfer (6e^−^ transfer),^[^ ^20^, ^24^ ^]^ including slow kinetics, complex intermediate adsorption/desorption, and high overpotential. By exploiting UOR, we could utilize its potential as an energy carrier and mitigate its environmental impact. Until now, the state‐of‐the‐art catalysts for urea electrolysis are noble metals, whereas their prohibitive cost, scarce reserves, and poor stability hinder widespread application. So, developing efficient, cheap, and robust UOR catalysts is highly urgent.

Recently it has been reported that several synthesis approaches and material design strategies are suitable for producing highly efficient UOR catalysts. The optimization of advanced catalyst performance involves various aspects such as porosity development, heterostructure design, defect engineering, surface functionalization, electronic structure modulation, and overall modifications of their structural and chemical properties.^[^ ^25^ ^]^ Moreover, nowadays there is a strong emphasis on researching nonprecious transition metal‐based catalysts, particularly Ni, Co, Mn, and Fe‐based catalysts, which show promising performance in UOR and OER.^[^ ^11^, ^26^, ^27^ ^]^ Catalysts such as CoSe_2_/NiSe_2_,^[^ ^28^ ^]^ Ni(OH)_2_,^[^ ^29^ ^]^ Ni(OH)_2_/SnS_2_,^[^ ^30^ ^]^ MoS_2_/Ni_3_S_2_,^[^ ^31^ ^]^ Ti‐doped Fe_2_O_3,_^[^ ^32^ ^]^ have been widely studied and have demonstrated excellent properties.^[^ ^20^, ^33^, ^34^, ^35^, ^36^ ^]^

Iron‐based catalysts hold great potential due to their earth‐abundance and low toxicity.^[^ ^16^ ^]^ Yet, the poor electronic conductor nature of iron‐based catalysts results in the formation of Schottky barriers between the catalyst and the electrolyte, as well as between the catalyst and the current collector, leading to sub‐optimal catalytic activity in these compounds.^[^ ^37^, ^38^ ^]^ These limitations can be addressed through elemental doping, which is a universal strategy to engineer the electronic and chemical properties of materials. This approach modifies the electronic structure of catalysts, creating new surface active sites and expediting the charge transfer.^[^ ^39^ ^]^ Various studies have suggested that pseudobrookite Fe_2_TiO_5_ can be used as light‐harvesting materials in PEC devices and as surface coatings to provide corrosion resistance.

In this work, we introduce a new active site with strong interaction with O to construct the dual active centers which may be an effective strategy to break adsorption‐energy scaling limitations, and thus improve the UOR activity.^[^ ^40^, ^41^, ^42^ ^]^ The Ru^4+^ with the electronic configuration of [Kr]4d has been reported to be a suitable adsorption site of H_2_O, OH, and N_2_ to participate in alkaline UOR and OER.^[^ ^43^ ^]^

Herein, we successfully introduce Ru dopants in Fe_2_TiO_5_, via a hydrothermal and CVD process for electrocatalytic hydrogen production via water and urea electrolysis. The obtained product exhibited superior activities and long‐term durability toward UOR and OER. When used as a UOR and OER electrocatalyst, low electrode potentials of 1.30 V and 1.40 V could be achieved to reach a current density of 10 mA cm^−2^ in 1.0 m KOH aqueous solutions with the absence and presence of 0.5 M urea, respectively. Also, density functional theory (DFT) simulations confirm that the charge distribution from Ru to Fe_2_TiO_5_ at the heterojunction's interfaces is advantageous for decomposing CO(NH_2_)_2_ and optimizing and accelerating UOR and OER.^[^ ^7^ ^]^

## 2 Experimental Section

### 2.1 Theoretical Simulations

**Figure 1:**
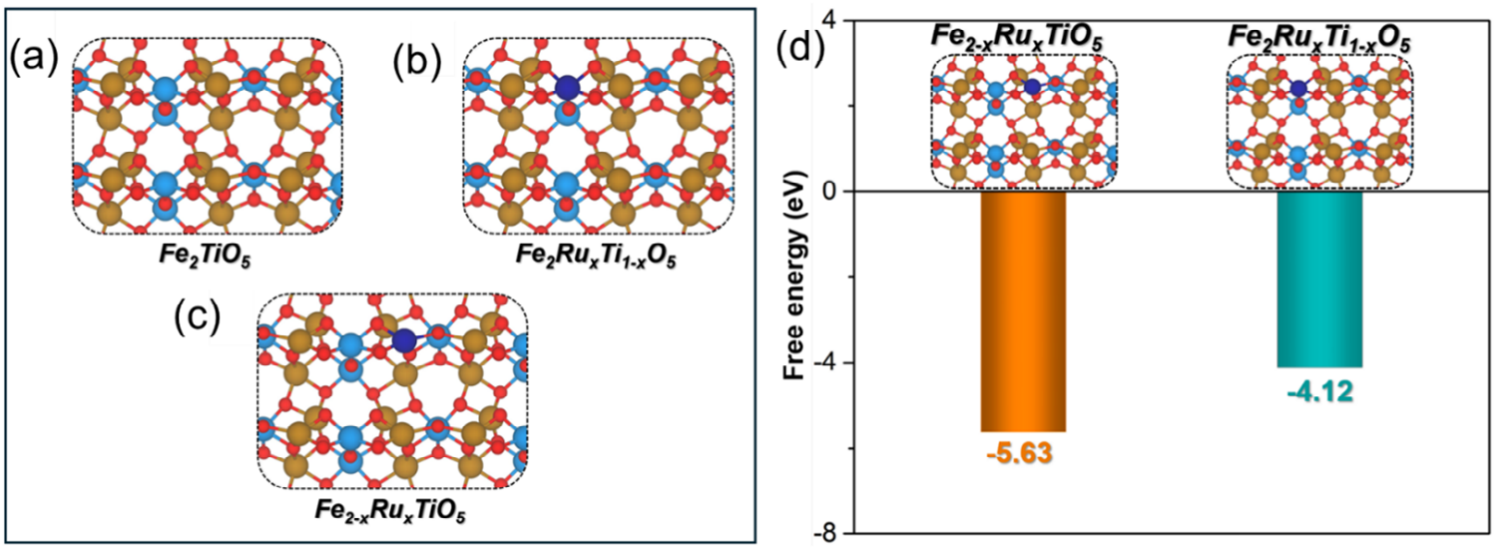
Catalyst surface models are investigated in this work. a) Fe_2_TiO_5_, b) Fe_2_Ru_x_Ti_1‐x_O_5_, c) Fe_2−x_Ru_x_TiO_5_. d) Formation energy of Fe_2_Ru_x_Ti_1‐x_O_5_ and Fe_2−x_Ru_x_TiO_5_ from Fe_2_TiO_5_.

To gain an understanding of the effect of Ru doping on the performance of the pristine Fe_2_TiO_5_ catalyst, first, the most stable phase was computationally studied when Ru substitutes Ti atoms on the surface, forming Fe_2_Ru_x_Ti_1‐x_O_5_, or substitutes Fe atoms, forming Fe_2−x_Ru_x_TiO_5_. To understand this phenomenon, Spin‐polarized DFT+U calculations were performed using the revised Perdew‐Burke‐Ernzerh of (RPBE) exchange‐correlation functional^[^ ^44^ ^]^ within the Vienna Ab Initio Simulation Package (VASP).^[^ ^45^ ^]^ The projector‐augmented wave (PAW) method^[^ ^46^, ^47^ ^]^ with a plane‐wave kinetic energy cutoff of 500 eV was utilized, along with Gaussian smearing of 0.05 eV. The DFT+U correction was applied with U values of 3 eV for Fe and 3.2 eV for Ti.^[^ ^48^, ^49^ ^]^ The Brillouin zone was sampled using a 3 × 3 × 1 k‐point mesh for geometry optimization and a 6 × 6 × 1 k‐point mesh for projected density of states (PDOS) calculations. The Fe_2_TiO_5_ (101) surface was modeled using a (3 × 3) supercell with a 15 Å vacuum layer to prevent periodic interactions. The Ru‐doped Fe_2_TiO_5_ was modeled as shown in **Figure** **1**a–c. All atoms were relaxed during geometry optimization until the forces were less than 0.03 eV/Å. The isolated molecules (O_2_, H_2_, H_2_O, N_2_, CO_2_, and CO(NH_2_)_2_) were optimized within a unit cell of 15 Å × 15 Å × 15 Å using only the Γ‐point.^[^ ^50^ ^]^ Van der Waals corrections were incorporated using Grimme's method with Becke–Johnson damping.^[^ ^51^ ^]^

The oxygen evolution reaction (OER) performance of Fe_2_TiO_5_(101) and Ru‐doped models was predicted using Gibbs free‐energy (ΔG) profiles for the following elementary OER steps, according to Norskov et al.:^[^ ^52^ ^]^

(1) StepI:OH−+∗→∗OH+e−

(2) StepII:∗OH+OH−→∗O+H2O+e−

(3) StepIII:∗O+OH−→∗OOH+e−

(4) StepIV:∗OOH→∗+O2+e−

where * represents the bare site and *OH, *O, *O_2,_ and *OOH denote the surface featuring different chemisorbed species. The free energy difference for all of the above elementary steps (ΔGOH*, ΔGO*, ΔGOOH*) involving an electron transfer is calculated by the equation Δ*G*  =  Δ*E* +  Δ*ZPE*  −  *T*Δ*S* +  Δ*G* *U* +  Δ*G* *pH*, where ΔE, ΔZPE, and ΔS correspond to the energy difference between adsorption energy, zero‐point energy, and entropy, respectively. The adsorption energies ΔE were measured using DFT. The ΔZPE and TΔS values were obtained from vibrational frequency calculations and DFT. ΔGU = −eU, where U represents a potential based on a standard hydrogen electrode. ΔGpH describes the Gibbs free energy correction of the pH, noting that it considers pH 12.

Under ideal conditions, the OER reaction with a total energy change of 4.92 eV can be driven at 1.23 V. In comparison, the free energy of each elementary reaction would be equally divided into 1.23 eV. Thus, the overpotential *η* is introduced to represent the additional required potential and to rationalize the catalytic performance of the catalyst, which is defined in theoretical calculations as: *η* = max(ΔG_(1,2,3,4)_)/e – 1.23 eV.

The overall reaction equation of urea oxidation (UOR) is presented as follows:

(5) CO(NH)2+6OH−→N2+5H2O+CO2+6e−

where the specific elementary steps are considered according to the following scheme:

(6) StepI:CO(NH2)2+∗→∗CO(NH2)2

(7) StepII:∗CO(NH2)2+OH−→∗CO(NH2NH)+H2O+e−

(8) StepIII:∗CO(NH2NH)+OH−→∗CO(NHNH)+H2O+e−

(9) StepIV:∗CO(NHN)+OH−→∗CO(NN)+H2O+e−

(10) StepV:∗CO(NN)→∗CO+N2

(11) StepVI:∗CO+OH−→∗COOH+e−

(12) StepVII:∗COOOH+OH−→∗CO2+H2O+e−

(13) StepVIII:∗CO2→∗+CO2

### 2.2 Synthesis of the Electrocatalyst

#### 2.2.1 Materials

These reagents were used without further purification: iron(III) nitrate nonahydrate (Fe (NO_3_)_3_, Merck *≥* 99.95%), isopropanol ((CH_3_)_2_CHOH, Merck *≥* 99.5%), titanium(IV) isopropoxide (Ti (OCH(CH_3_)_2_)_4_, Merck %97), ruthenium(III) chloride nonahydrate (RuCl_3_*9H_2_O, Merck 45–55%), absolute ethanol (CH_3_CH_2_OH), deionized water (H_2_O).

Synthesis of pseudobrookite Fe_2_TiO_5_: To synthesize the pseudobrookite Fe_2_TiO_5_, 2.51 mmol of Fe (NO_3_)_3_.9H_2_O were dissolved in 50 mL of isopropanol under stirring. Then, 1.25 mmol of Ti (OCH(CH_3_)_4_) were added dropwise to the solution. The resulting mixture underwent stirring for 1 hr. Then, the solution was transferred to a Teflon‐lined stainless‐steel autoclave and subjected to a solvothermal treatment at 150 °C for 12 hrs, with a controlled heating rate of 10 °C min*^−^* ^1^. After cooling, the suspension was centrifuged and washed with deionized water and isopropanol three times. The powder was then dried in a 60 °C oven for 12 h, then calcinated in a furnace at 800 °C for 3 h.

Synthesis of Fe_2−x_Ru_x_TiO_5_: In a typical process of Fe_2−x_Ru_x_TiO_5_ synthesis (Scheme S1), 5.01 mmol Fe_2_TiO_5_, and 0.1082 mmol RuCl_3_·9H_2_O were dissolved in 10 mL of ethanol under stirring for 3 hrs. Later, 20 ml of deionized water were added to the solution and stirred for 3 h. The entire solution was then transferred to a Teflon‐lined stainless‐steel autoclave and subjected to a thermal treatment at 150 °C for 12 h. After natural cooling, the resulting sample was centrifuged and dried for 12 h at 60 °C, followed by calcination at 300 °C for 2 h under Ar atmosphere.

### 2.3 Material Characterization Techniques

The samples' crystalline structure and phase composition were examined using X‐ray diffraction (XRD) on a TZY‐XRD (D/MAX‐TTRIII(CBO)) system with Cu–Kα radiation (λ = 1.5418 Å). The morphology and microstructure were characterized through Scanning Electron Microscopy (SEM) on a Hitachi S4800 field‐emission scanning electron microanalyzer coupled with energy‐dispersive X‐ray spectroscopy (EDX). High‐resolution Transmission Electron Microscopy (HR‐TEM) and High Angle Annular Dark Field – Scanning Transmission Electron Microscopy (HAADF‐STEM) micrographs were also recorded via Nancy JEOL ARM 200F with 2 CS correctors. The TEM has an in Nancy: MSC794 camera Gatan one view, JEOL HAADF detector, and a double‐tilt sample holder. X‐ray photoelectron spectroscopy (XPS) measurements were carried out in a PHI 5700 Physical Electronics spectrometer, with non‐monochromatic Mg Kα radiation equipped with a multichannel detector. X‐ray absorption spectroscopy (XAS) measurement at Fe K‐edge was carried out at the XAFS experimental end station of the synchrotron Elettra (Andrea Di Cicco et al., https://doi.org/10.1088/1742‐6596/190/1/012043) by mounting the sample (as pellets) on a sample holder using Kapton tape in a vacuum chamber. The Si (1 1 1) fixed‐exit double crystal monochromator of 1.4 × 10^−4^ resolving power was calibrated to the first‐derivative maximum of the K‐edge absorption spectrum of a metallic Fe foil (7112.0 eV) and it was detuned to exclude higher order harmonics.

### 2.4 Electrochemical Measurements

Typically, the electrochemical performance was measured using a Biologic electrochemical workstation instrument with techniques like Linear scanning voltammetry (LSV), cyclic voltammetry (CV), chronopotentiometry (CP), and Electrochemical impedance spectroscopy (EIS). All the electrochemical experiments were carried out in a three‐electrode set‐up (Ag/AgCl‐reference, Pt wire‐counter, and as‐prepared catalysts‐working electrode, electrolyte‐ 1.0 m KOH). Before measuring LSV, CV was performed at a scan rate of 50 mV s^−1^ for 20 cycles to activate the surface of the catalyst. Then, LSV was carried out at a scan rate of 1 mV s^−1^. The electrochemical double‐layer capacitance (C_dl_) of various electrocatalysts was also obtained using the CV in the non‐Faradic area at varied sweeping speeds spanning from 180 mV s^−1^ to 260 mV s^−1^. Electrochemical impedance spectroscopy (EIS) measurements were carried out from 0.1 to 100 000 Hz with an amplitude of 5 mV and an overpotential of 0.3 V. The electrochemical double‐layer capacitance, expected to be linearly proportional to the Electrochemical active surface area (ECSA), was determined by measuring the capacitive current at non‐Faradic regions from scan rate‐dependent CV runs.

## 3 Results and Discussion

### 3.1 Theoretical Insights

The DFT calculations predicted the potential substitution sites for Ru in the Fe‐Ti‐O system. Ru could either replace Ti, forming Fe_2−x_Ru_x_TiO_5_, or Fe, resulting in Fe_2−x_Ru_x_TiO_5_. To resolve this ambiguity, the formation energies for both scenarios were calculated. Figure 1 presents the calculated formation energies for Fe_2_Ru_x_Ti_1−x_O_5_ and Fe_2−x_Ru_x_TiO_5_. The results indicate that the formation of Fe_2−x_Ru_x_TiO_5_ is slightly more thermodynamically favorable than that of Fe_2_Ru_x_Ti_1−x_O_5_, suggesting that Fe_2−x_Ru_x_TiO_5_ may be the dominant phase in the real catalyst. However, the possible existence of Fe_2_Ru_x_Ti_1−x_O_5_ cannot be excluded, prompting us to investigate the activity of both models toward the OER and UOR to better understand the active sites. Inspired by this, we experimentally produced the predicted systems.

### 3.2 Morphology, and Crystal Structure Characterizations

**Figure 2:**
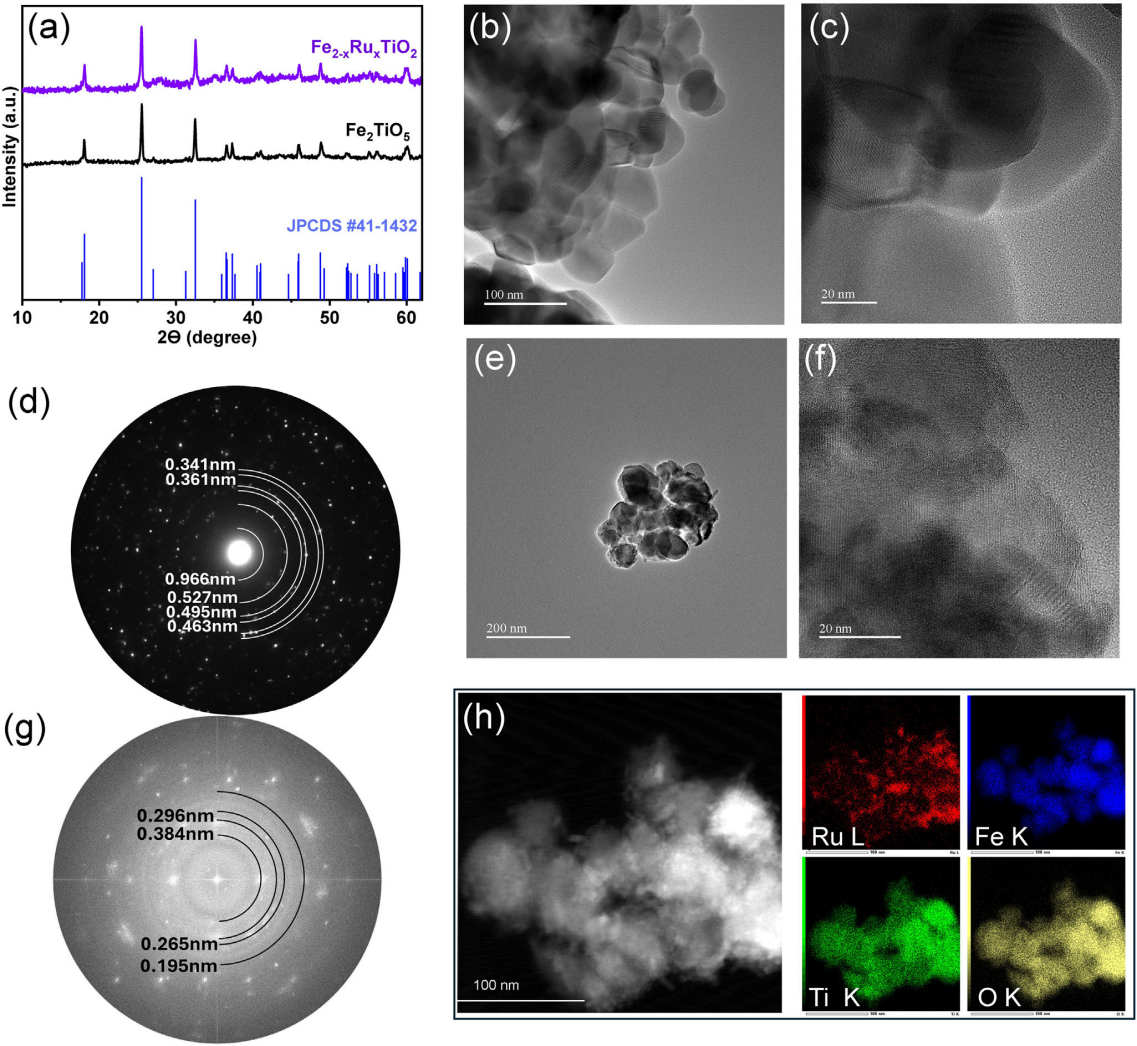
a) XRD, b) TEM image, c) HRTEM image d), SAED pattern of Fe_2_TiO_5_, e) TEM image, f) HRTEM image, g) SAED pattern of Fe_2−x_Ru_x_TiO_5_, h) HAADF_STEM and corresponding elemental mapping of Ru, Fe, Ti, and O in Fe_2−x_Ru_x_TiO_5_.

The pure Fe₂TiO₅ pseudobrookite crystal phase with an orthorhombic space group, Bbmm (63), was achieved at 800 °C for 3 h and characterized by XRD. As depicted in **Figure** **2**a, the diffraction reflections appeared at 18.1°, 25.5°, 32.4°, 36.5°, 37.4°, 45.9°, 48.7°, and 59.8°, corresponding to the (200), (101), (230), (301), (131), (430), (020), and (232) planes of Fe₂TiO₅. All peaks in the diffraction pattern agree with the standard PDF #41‐1432, confirming the formation of the pure Fe₂TiO₅ phase. To introduce Ru doping, different amounts of Ru precursor (0.04, 0.06, 0.08, and 1.0g) were considered to optimize the appropriate Ru content that could be incorporated without inducing an additional crystal phase in Fe₂TiO₅, aiming for the formation of _2−x_Ru_x_TiO_5_. This was synthesized using hydrothermal synthesis followed by CVD treatment. The pure _2−x_Ru_x_TiO_5_ phase was achieved at x = 0.04 Ru content. XRD characterization (Figure 2a) of the Ru‐doped material revealed reflection peaks like Fe₂TiO₅, with a slight shift of the (101) diffraction peak to a lower angle. This shift indicates an increase in interplanar spacing, suggesting the successful incorporation of Ru⁴⁺ ions into the Fe₂TiO₅ structure. However, as shown in Figure S1a (Supporting Information), when x exceeds 0.04, Ru tends to aggregate and form RuO₂ instead of incorporating into the Fe₂TiO₅ lattice. This leads to the formation of a RuO₂‐Fe₂TiO₅ heterostructure rather than true Ru doping. As illustrated in Figure 2b, the TEM analysis reveals that Fe₂TiO₅ consists of nanoparticles, with a mean particle size of ≈52 nm, as estimated from Figure S1b (Supporting Information) and further supported by Figure S1c (Supporting Information). The HRTEM images (Figure 2c) display that the nanoparticles are highly crystalline: crystal lattice spacing is observed and the corresponding SAED pattern (Figure 2d) shows a clear diffraction pattern. The marked crystal lattice spacing is 4.56, and 3.34 Å, corresponding to (111) and (220) crystallographic planes of Fe_2_TiO_5_ (PDF #41‐1432), which is consistent with XRD analysis and the standard patterns of Fe_2_TiO_5_ (PDF #41‐1432). When Ru was incorporated, the TEM image (Figure 2e) demonstrated that smaller particles (less than ≈49 nm, Figure S1c, Supporting Information) could be observed on Fe_2−x_Ru_x_TiO_5_. With Ru loading, the HRTEM image (Figure 2f) and SAED pattern (Figure 2g) display diffraction spots at 2.88, 4.94, and 3.43 Å, corresponding to (121), (200), and (101) crystallographic planes of Ru doped Fe_2_TiO_5_ structure, respectively. HAADF_STEM images (Figure 2h) and corresponding elemental mapping confirm the presence and spatially homogeneous distribution of Ru, Fe, Ti, and O elements uniformly dispersed in the Fe_2−x_Ru_x_TiO_5_.

**Figure 3:**
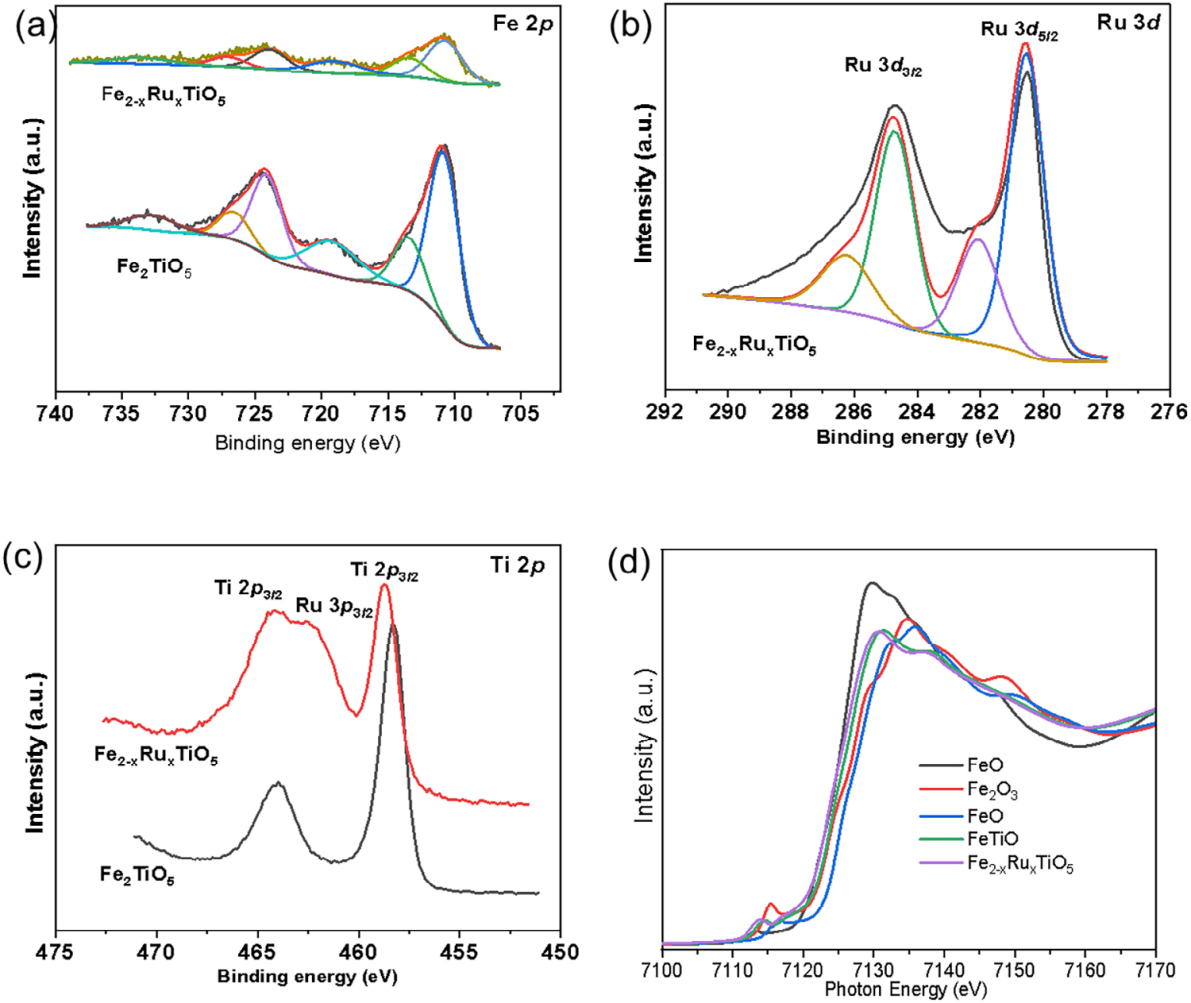
High‐resolution XPS spectra of Fe_2_TiO_5_ and Fe_2−x_Ru_x_TiO_5_ a) Fe 2*p* b) Ti 2*p* c) Ru 3*d* and d) O 1s. e) XAS Fe K‐edge spectra for FeO, Fe_2_O_3_, Fe_3_O_4_, Fe_2_TiO_5_ and Fe_2−x_Ru_x_TiO_5_.

XPS analysis was used to understand the samples' surface chemistry and oxidation states. Figure S2a,b (Supporting Information) shows the survey XPS spectra for Fe₂TiO₅ and _2−x_Ru_x_TiO_5_ samples, which affirm the presence of all the expected elements, that is, Ti, Fe, O for Fe₂TiO₅, and the addition of Ru in the Fe_2−x_Ru_x_TiO_5_. The deconvoluted high‐resolution Fe 2*p* core level spectrum of Fe_2_TiO_5_ (**Figure** **3**a), shows contributions at 724.1 and 710.8 eV assigned to the doublet Fe 2*p*_1_ *_/_*_2_ and Fe 2p_3_ *_/_*_2_, respectively, indicating Fe^3+^ with a 13.3 eV gap. Deconvolution revealed a contribution at 713.4 eV, and satellite peaks at 719.1 and 732.7 eV that confirm the oxidation state of Fe^3+^. In the Fe_2−x_Ru_x_TiO_5_ sample, the high‐resolution Fe 2*p* core level spectrum showed a slight shift of 0.2 eV toward lower binding energies, suggesting a possible electron density redistribution between Ru and Fe at the surface region.

The BE of Ti 2*p* in Fe_2_TiO_5_ appeared at ≈458.3 and 463.9 eV, indicative of Ti^4+^ oxidation states, as shown in Figure 3b. Upon Ru doping, the Ti 2*p*_3/2_ binding energy shifts to a higher energy of 458.7 eV, specifically by 0.4 eV, pointing out the electron density redistribution between Ru and Fe at the surface region. However, part of the doublet Ti 2*p*_1/2_ is masked by the Ru 3*p*_3/2_ signal. To further validate the oxidation state of Ti, we examined the Ti 3*p* spectrum (Figure S2c, Supporting Information), which confirmed the presence of Ti (IV) ions, octahedrally coordinated by oxygen ions. As depicted in Figure 3c the Ru 3*d* peaks at 282.1 eV and 286.0 eV confirmed the presence of Ru^4+^. Due to the Ru 3d signal overlapping with the C 1*s* signal, the Ru 3*p*_1/2_ at 488.1 eV (Figure S2d, Supporting Information) was measured, further confirming the presence of Ru^4+^ in Fe_2−x_Ru_x_TiO_5_. However, peaks at 484.7 and 280.5 eV in both Ru 3*p* and Ru 3d spectra indicated the presence of unreacted Ru (0) on the surface. Finally, the high‐resolution O 1*s* core level spectra (Figure S2e, Supporting Information) were resolved into two contributions: one at 529.9 eV, associated with lattice oxygen, and another ≈ 531.4 eV, attributed to Fe‐OH groups in both doped and undoped samples. Hence, the combined study shows that the nano‐structured material and Ru doping were successfully synthesized. To further explore the electronic structure and crystal structure distortion, Fe K‐edge XAS spectra (Figure 3e) were collected for both Fe₂TiO₅ and Fe_2−x_Ru_x_TiO_5_ and compared with reference spectra from FeO, Fe₂O₃, and Fe₃O₄. The alignment of the Fe K‐edge spectrum of Fe₂TiO₅ with that of Fe₂O₃ confirms the presence of Fe^3^⁺. However, after Ru doping, the main edge shifts to lower photon energy, aligning with FeO, which indicates a partial reduction of Fe in Fe_2−x_Ru_x_TiO_5_ toward the Fe^2^⁺ state. This observation is consistent with our Fe 2p XPS results. Additionally, the change in pre‐edge peak intensity in the Fe K‐edge indicates a local structure distortion at the Fe site, further supported by our DFT calculations. This suggests that Ru substitutes the Fe site to form Fe_2−x_Ru_x_TiO_5_. Our findings align closely with the results reported in earlier research studies.^[^ ^53^, ^54^ ^]^ This consistency suggests that our outcomes support the validity of the conclusions drawn by prior investigators.

### 3.3 Electrochemical Evaluation of UOR

**Figure 4:**
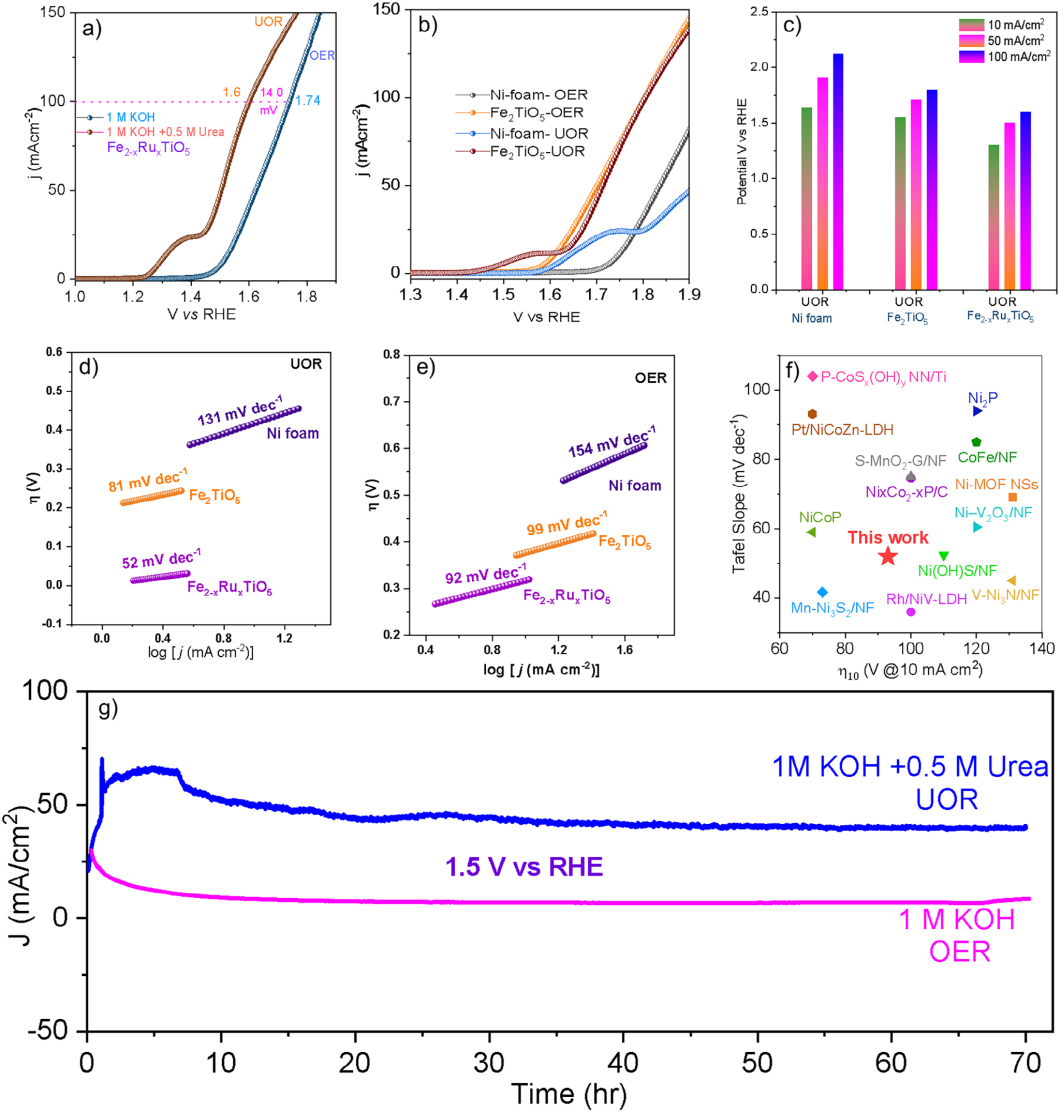
a) Polarization curves of Fe_2−x_Ru_x_TiO_5_ electrocatalysts in 1.0 m KOH with and without 0.5 m urea. b) LSV curves of NF and Fe_2_TiO_5_. c) Comparison of UOR potentials at 10, 50, and 100 mA cm^−2^ current densities. Tafel plots for d) UOR and e) OER. f) Comparison of the UOR overpotential of Fe_2−x_Ru_x_TiO_5_ at a current density of 10 mA cm^−2^ with other reported works. g) Chronoamperometric run of Fe_2−x_Ru_x_TiO_5_ for the long‐term durability test at 1.5V.

The electrocatalytic performance of the designed samples for UOR was investigated using a standard three‐electrode cell in a 1.0 m KOH solution with 0.5 m urea. **Figure** **4**a shows the OER electrocatalytic performance of Fe_2−x_Ru_x_TiO_5_ and Fe_2_TiO_5_. Specifically, in 1 m KOH the potential required by Ni foam, Fe_2_TiO_5_, and Fe_2−x_Ru_x_TiO_5_ for OER to reach a current density of 10 mA cm^−2^ is 1.75,1.61 and 1.50 V. However, with 1 M KOH +0.5M urea, the potential at 100 mA cm^−2^ (E_100_) of this catalyst can reach 1.6 V, ≈ 140 mV lower than that in the pure KOH solution, implying that the UOR is more energy‐efficient than the OER for Fe_2−x_Ru_x_TiO_5_. The Fe₂TiO₅ catalyst requires potentials of 1.61, 1.70, and 1.80 V to achieve current densities of 10, 50, and 100 mA cm^−^^2^ for the OER (Figure S4a, Supporting Information). However, after Ru doping, Fe_2−x_Ru_x_TiO_5_ shows a significant reduction in the required potentials, needing only 1.50, 1.62, and 1.74 V for the same current densities. A similar trend is observed under UOR conditions (Figure 4c), where Fe_2−x_Ru_x_TiO_5_ achieves 10, 50, and 100 mA cm^−^^2^ at 1.29, 1.50, and 1.60 V, respectively, in contrast to Fe₂TiO₅, which requires 1.55, 1.71, and 1.80 V. The significant change in potential in Ru doped sample and under the presence of Urea reflects that Ru doping and urea play a crucial role in enhancing catalytic performance. For a fairer comparison that would eliminate the influence of the catalyst's surface area and focus on its true intrinsic activity, we normalized the data with ECSA.^[^ ^55^ ^]^ As can be seen from Figure S4b (Supporting Information), a similar trend is observed which illustrates that electrocatalytic performance emanates from the intrinsic behavior.

To better understand the UOR and OER kinetics, the Tafel slopes for doped and undoped samples were determined. As a result, Fe_2−x_Ru_x_TiO_5_ displays a remarkably smaller Tafel slope of 52 mV dec^−1^ in UOR (Figure 4d) compared to the Tafel plot of 92 mV dec^−1^ in OER (Figure 4e), indicating a rapid current density increase and favorable kinetics for UOR. The performance and kinetics of Fe_2−x_Ru_x_TiO_5_ for UOR were also compared with literature reports, demonstrating superior activity and fast kinetics surpassing the most recently reported works, as shown in Figure 4f. To investigate the reasons for the high UOR activity of Fe_2−x_Ru_x_TiO_5_, we first calculated its double‐layer capacitance (*C*_dl_), which is proportional to the electrochemical active surface area (ECSA). To unveil this, we calculated the C_dl_ by monitoring the current density in the non‐Faradic region with different scan rates. The CV run for obtaining these data is presented in Figure S3c–f (Supporting Information). As a result, Figure S4a,b (Supporting Information) shows the Cdl value of Fe_2_TiO_5_ (27.7 mF cm^−2^, UOR and 30.0 mF cm^−2^, OER) and Fe_2−x_Ru_x_TiO_5_ (33.0 mF cm^−2^, UOR and 35.8 mF cm^−2^, OER), respectively. The UOR Cdl value for Ru doping of Fe_2−x_Ru_x_TiO_5_ is 2.7 times higher than that of Fe_2_TiO_5_, demonstrating that Ru doping can greatly improve the UOR intrinsic activity. Moreover, the EIS at the solid−liquid interface was measured to study electrical conductivity. As shown in Figure S4c (Supporting Information), Fe_2−x_Ru_x_TiO_5_ exhibits a charge transfer resistance (Rct) of only 0.91Ω, lower than Fe_2_TiO_5_(1.02 Ω) under UOR. Similarly, under OER, Fe_2−x_Ru_x_TiO_5_ shows an Rct (Figure S4d, Supporting Information) of only 1.75 Ω, again lower than that of Fe_2_TiO_5_(1.78 Ω), implying the enhanced electron transfer rate in Fe_2−x_Ru_x_TiO_5_.

In addition to catalytic activity, stability is another fundamental criterion for evaluating catalysts. Herein, we examined the Chronoamperometric performance (Figure 4g) of the catalyst at a potential of 1.5 V over an extended period of 72 hr with negligible potential change, signifying the robust OER and UOR stability. We also examined the Chronopotentiometric performance (Figure S4e, Supporting Information) at fixed current densities (10, 50, and 150 mA cm*^−^* ^2^) over an extended period of 36 hr. At lower current densities of 10 and 50 mA cm*^−^* ^2^, the catalyst exhibited stable potentials for both OER and UOR. However, at a higher current density of 150 mA cm*^−^* ^2^, a slight drop in potential was observed only in OER, with no corresponding change observed in the UOR counterpart. It is noteworthy that the increase in potential during OER occurred primarily in the final hours, while the potential remained relatively constant during the initial hours. The long‐term stability for Fe_2−x_Ru_x_TiO_5_ under KOH and KOH + urea also compares fairly with the most recently reported literature, as displayed in Table S1 (Supporting Information). This impressive resilience suggests the potential for long‐term practical applications of the catalysts.

Meanwhile, after drop‐casting Fe_2−x_Ru_x_TiO_5_ onto Ni foam, post‐UOR XRD analysis was conducted to evaluate the catalyst's structural stability. Although the intense peaks from the Ni foam obscure some characteristic peaks of our material, an enlarged peak (Figure S5, Supporting Information) is still observed at 25.5° and 32.4°, corresponding to the (101) and (230) planes of Fe_2−x_Ru_x_TiO_5_. This confirms the material's stability even after long‐term operation.

### 3.4 Mechanism of Electrocatalytic Urea Oxidation Reaction

**Figure 5:**
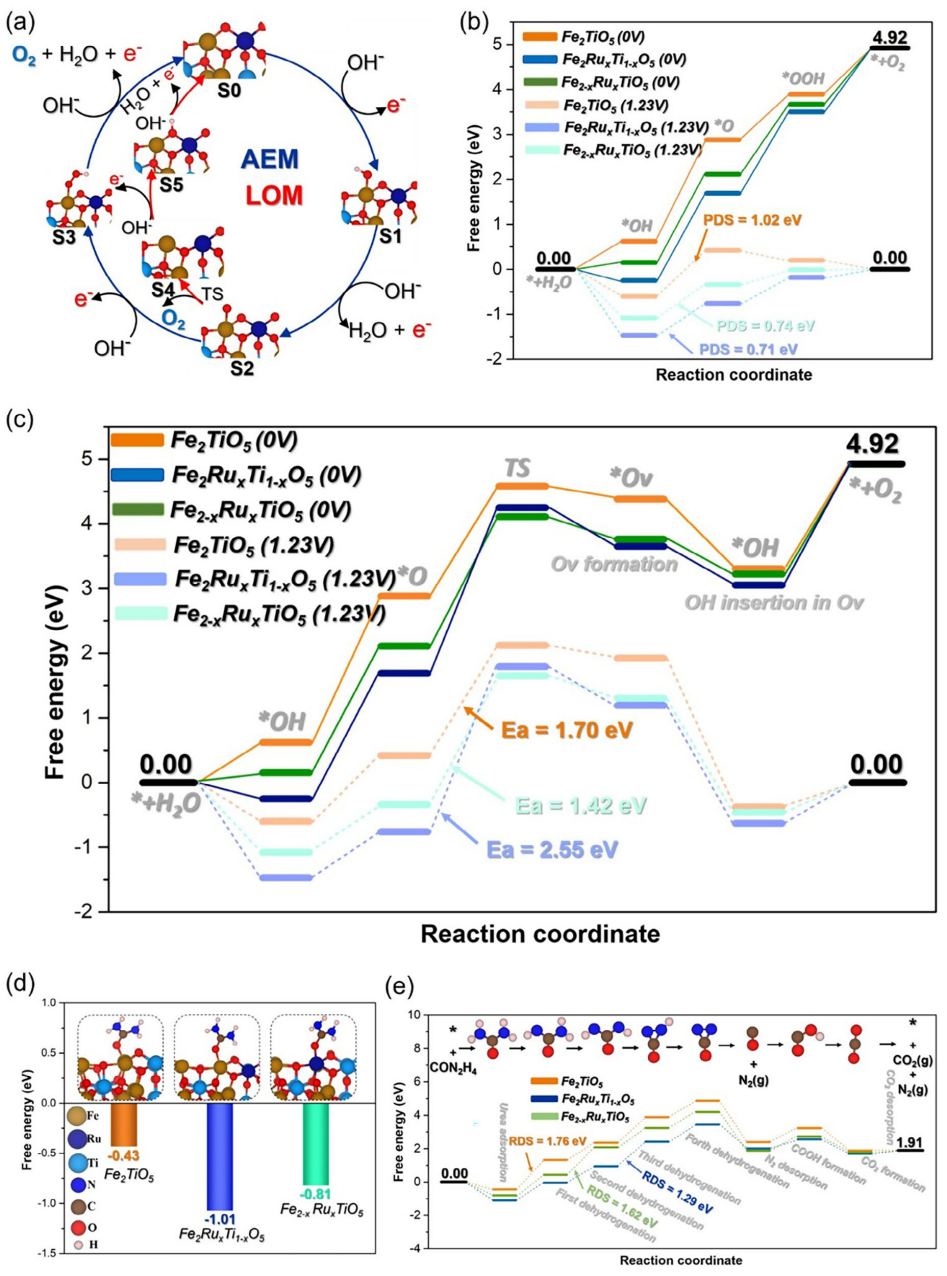
a) OER mechanism pathways via AEM and LOM on Fe_2_RuxTi_1‐x_O_5_. b) OER energy profile on Fe_2_TiO_5_, Fe_2_Ru_x_Ti_1‐x_O_5_, and Fe_2−x_Ru_x_TiO_5_ via AEM mechanism calculated at 0 V and 1.23 V. c) OER energy profile on Fe_2_TiO_5_, Fe_2_Ru_x_Ti_1‐x_O_5,_ and Fe_2−x_Ru_x_TiO_5_ via LOM mechanism calculated at 0 and 1.23 V. d) Urea adsorption energy on different catalyst models Fe_2_TiO_5_, Fe_2_Ru_x_Ti_1‐x_O_5_ and Fe_2−x_Ru_x_TiO_5_. e) Urea oxidation energy profile on Fe_2_TiO_5_, Fe_2_Ru_x_Ti_1‐x_O_5,_ and Fe_2−x_Ru_x_TiO_5_.

Experimental efforts unveiled that the rationally synthesized Fe_2−x_Ru_x_TiO_5_ holds superior catalytic activity and robust stability. To further understand the reaction mechanism and the underlying reason for the higher activity, density functional theory (DFT) simulations were conducted. Given the nature of the catalyst surface, we proposed two possible mechanisms to investigate the OER reaction: the adsorbate evolution mechanism (AEM), based on the evolution of adsorbed intermediates, and the lattice oxygen mechanism (LOM), where lattice oxygen could be involved in the OER process, as depicted in **Figure** **5**a. Similarly, Figure 5b presents the OER energy profile for the three models using the AEM mechanism. The data show that the Fe_2_TiO_5_ catalyst has a high energy barrier of 2.25 eV, with a potential‐limiting step of 1.02 V. This suggests that OER processing on the Fe_2_TiO_5_ surface is challenging at low electrode potentials, with the rate‐determining step being the transformation of *OH to *O. The introduction of a Ru atom in place of Ti reduces the barrier energy to 1.94 eV, and the potential‐determining step decreases to 0.71 V, still associated with the *OH to *O transformation. A similar activity is observed when Ru substitutes for Fe, with a barrier energy of ≈1.97 eV and an overpotential of ≈0.74 V. In all three models, the rate‐determining step is the *OH to *O transformation, which can be attributed to the high oxidation environment of the Fe_2_TiO_5_ catalyst and its Ru‐doped counterpart. This high oxidation environment necessitates a significant energy barrier for the formation of the *O intermediate, which is moderated by the introduction of Ru atoms at the Ti or Fe sites.

Due to the high oxidation environment of the catalyst models, we also investigated the LOM mechanism, exploring the possibility of lattice oxygen involvement in O_2_ production via association with the *O intermediate. The energy profile depicted in Figure 5c shows that the associative process of the *O intermediate with lattice oxygen to form O_2_ and create an oxygen vacancy requires high barrier energies. These barriers are ≈1.70, 2.55, and 1.42 eV for the Fe_2_TiO_5_, Fe_2_Ru_x_Ti_1‐x_O_5_, and Fe_2−x_Ru_x_TiO_5_ catalyst models, respectively, indicating a significant likelihood of occurrence under real operating conditions.

We next examine the UOR mechanism in the three models, beginning with an analysis of the urea adsorption energy as an initial step for subsequent oxidation. As illustrated in Figure 5d, urea exhibits the highest adsorption energy of ≈−1.01 eV on the Fe_2_Ru_x_Ti_1‐x_O_5_ surface, while the Fe_2−x_Ru_x_TiO_5_ surface shows a moderate adsorption energy of ≈−0.81 eV. In contrast, the pristine Fe_2_TiO_5_ surface demonstrates a relatively low adsorption energy of ≈−0.43 eV. The subsequent reaction pathway, detailed in Figure 5e, reveals that all three models adsorb urea more strongly than the *OH intermediate. This suggests that, under operational conditions, urea can preferentially occupy the catalyst's active sites due to its higher adsorption energy compared to OH^−^.

The initial step of urea activation is critical, as it determines the feasibility of urea decomposition. As shown in Figure 5e, the first step of urea dehydrogenation requires a barrier energy of 1.76 eV on the pristine Fe_2_TiO_5_ surface. In contrast, this barrier is significantly lower in the Ru‐doped models, requiring 1.12 eV on Fe_2_Ru_x_Ti_1‐x_O_5_ and 1.26 eV on Fe_2−x_Ru_x_TiO_5_. Notably, this first step is the rate‐determining step for the pristine Fe_2_TiO_5_ surface, but not for the doped models. For Fe_2_Ru_x_Ti_1‐x_O_5_, the rate‐determining step is the third dehydrogenation step, with a barrier energy of 1.29 eV. For Fe_2−x_Ru_x_TiO_5_, the second dehydrogenation step is rate‐limiting, with a barrier energy of 1.62 eV. These findings suggest that high adsorption of certain reaction intermediates and control of their adsorption energy and interactions with the catalyst surface can enhance urea oxidation.

**Figure 6:**
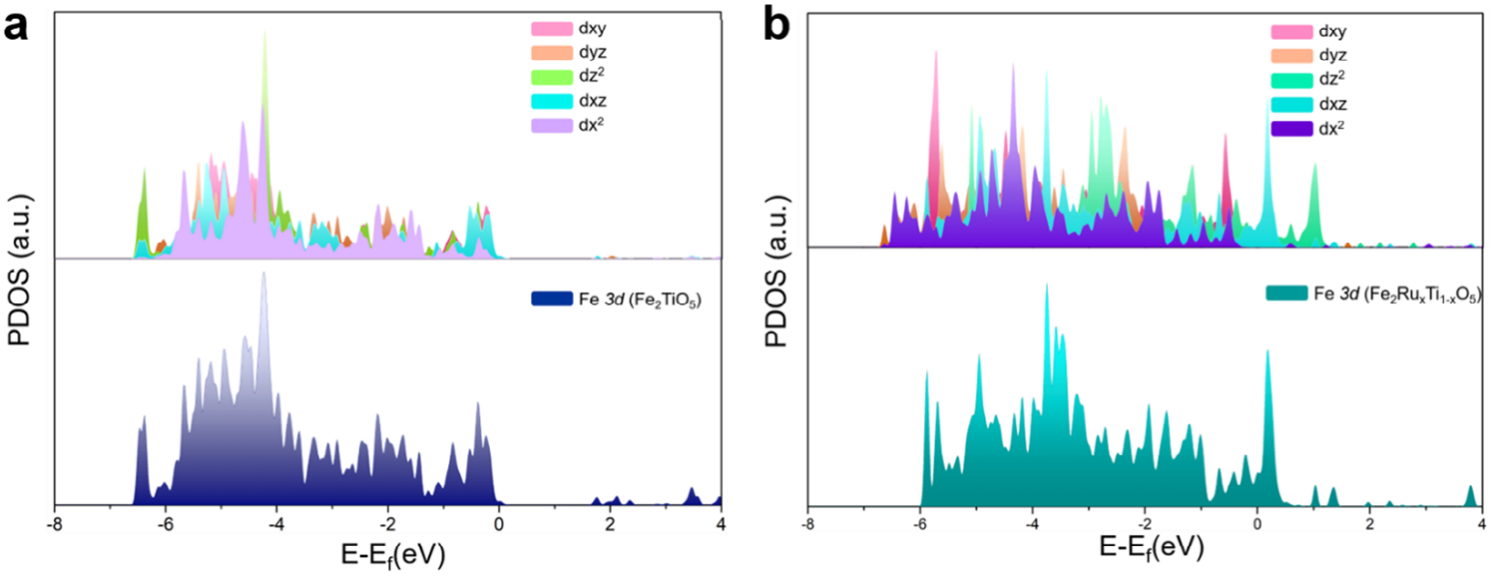
Partial density of states (PDOS) of d‐projected DOS of Fe active site in a) Fe_2_TiO_5_ and b) Fe_2_Ru_x_Ti_1‐x_O_5_.

To better understand the effect of Ru doping on the Fe active site in Fe_2_TiO_5_, we performed a projected density of states (PDOS) analysis, as shown in **Figure** **6**a,b. The results indicate that the introduction of Ru atoms shifts the Fe 3*d* orbitals upward, suggesting a reduction in the oxidation state of the Fe sites. This shift helps to explain the enhanced adsorption of reactants, including the OH intermediate for the OER reaction and urea. Notably, we also observed a splitting of the Fe 3*d* orbitals, with the *d*z^2^ and *d*xz orbitals shifting to higher energy levels. These results may also indicate a crystal structure distortion, consistent with observations from experimental results from the XPS Fe 2*p* spectra (Figure 3a) and Fe K‐edge XAS (Figure 3e).

## 4 Conclusion

In summary, Ru‐doped pseudobrookite Fe_2_TiO_5_ was successfully synthesized using a two‐step hydrothermal treatment. This approach yielded a sample with high crystallinity, well‐defined morphology, and precise ruthenium doping, as predicted by first‐principles calculations. Additionally, the resulting nanostructure demonstrated exceptional efficiency and stability as an electrocatalyst for both OER and UOR. Its low overpotential of 150 mV, a Tafel slope of 52 mV dec^−1^, and remarkable stability over 72 h make this material one of the most promising candidates in the UOR field. From the DFT simulation, Ru‐doped models showed higher urea adsorption energies and lower energy barriers for initial dehydrogenation steps to the pristine Fe_2_TiO_5_. The Ru doping donates electrons back at large overpotential and prevents Fe dissolution, as confirmed via first‐principle computation. The dynamic electron interaction between Ru and Fe facilitates the adsorbate evolution mechanism and lowers adsorption energies for oxygen intermediates to boost the activity and stability of Fe_2_Ru_x_Ti_1‐x_O_5_. This study paves the way for advancing water oxidation through innovative material development and using urea‐assisted water splitting, which collectively helps oxidation reduce water oxidation's kinetics.

## Conflict of Interest

The authors declare no conflict of interest.

## Author Contributions

K.B.I., M.H., K.H., and M.B. contributed equally to this work. A.V., K.B.I., and E.M. supervised the project. T.A.S. conceived and designed the project. P.S. and M.H. perform the ECSA and EIS measurements. K.H. carried out the theoretical calculation. S.B., D.H. TEM characterization and E. R‐C: XPS characterization assisted in material characterizations. D.O.D.‐S., and L.O. measure XAS, M.G.S., and M.B. wrote the manuscript in consultation with all the other authors.

## Supporting information



Supporting Information

## Data Availability

The data that support the findings of this study are available from the corresponding author upon reasonable request.

## References

[1] Z. Ji , J. Liu , Y. Deng , S. Zhang , Z. Zhang , P. Du , Y. Zhao , X. Lu , J. Mater. Chem. A 2020, 8, 14680.

[2] J. Liang , Z. Cai , Z. Li , Y. Yao , Y. Luo , S. Sun , D. Zheng , Q. Liu , X. Sun , B. Tang , Nat. Commun. 2024, 15, 2950. 38580635 10.1038/s41467-024-47121-x PMC10997793

[3] T. A. Shifa , A. Gradone , K. Yusupov , K. B. Ibrahim , M. Jugovac , P. M. Sheverdyaeva , J. Rosen , V. Morandi , P. Moras , A. Vomiero , Chem. Eng. J. 2023, 453, 139781.

[4] B. Zhao , J. Liu , C. Xu , R. Feng , P. Sui , J.‐X. Luo , L. Wang , J. Zhang , J.‐L. Luo , X.‐Z. Fu , Appl. Catal., B 2021, 285, 119800.

[5] X. Deng , M. Li , Y. Fan , L. Wang , X.‐Z. Fu , J.‐L. Luo , Appl. Catal., B 2020, 278, 119339.

[6] S. Hu , S. Wang , C. Feng , H. Wu , J. Zhang , H. Mei , ACS Sustainable Chem. Eng. 2020, 8, 7414.

[7] S. Wang , L. Zhao , J. Li , X. Tian , X. Wu , L. Feng , J. Energy Chem. 2022, 66, 483.

[8] Q. Qian , J. Zhang , J. Li , Y. Li , X. Jin , Y. Zhu , Y. Liu , Z. Li , A. El‐Harairy , C. Xiao , G. Zhang , Y. Xie , Angew. Chem., Int. Ed. 2021, 60, 5984. 10.1002/anie.202014362 33306263

[9] S. Liang , L. Pan , T. Thomas , B. Zhu , C. Chen , J. Zhang , H. Shen , J. Liu , M. Yang , Chem. Eng. J. 2021, 415, 128864.

[10] C. Wei , Z. J. Xu , Small Methods 2018, 2, 1800168.

[11] C. Li , Y. Liu , Z. Zhuo , H. Ju , D. Li , Y. Guo , X. Wu , H. Li , T. Zhai , Adv. Energy Mater. 2018, 8, 1801775.

[12] X. Gu , D. Yang , Z. Liu , S. Wang , L. Feng , Electrochim. Acta 2020, 353, 136516.

[13] A. S. Rasal , H. M. Chen , W.‐Y. Yu , Nano Energy 2024, 121, 109183.

[14] N. Chen , Y.‐X. Du , G. Zhang , W.‐T. Lu , F.‐F. Cao , Nano Energy 2021, 81, 105605.

[15] A. N. Rollinson , J. Jones , V. Dupont , M. V. Twigg , Energy Environ. Sci. 2011, 4, 1216.

[16] J. Li , S. Wang , J. Chang , L. Feng , Adv. Powder Mater. 2022, 1, 100030.

[17] J. Li , X. Xu , X. Hou , S. Zhang , G. Su , W. Tian , H. Wang , M. Huang , A. Toghan , Nano Res. 2023, 16, 8853.

[18] J. Xie , R. Ding , Y. Li , J. Guo , Y. Zhang , Q. Fang , M. Yan , Y. He , Z. Yan , Z. Chen , X. Guo , Q. Yang , J. Luo , Y. Zhang , X. Sun , E. Liu , Nano Energy 2024, 126, 109669.

[19] Y. Liu , D. Zheng , Y. Zhao , P. Shen , Y. Du , W. Xiao , Y. Du , Y. Fu , Z. Wu , L. Wang , Int. J. Hydrogen Energy 2022, 47, 25081.

[20] T. Yu , Q. Xu , J. Chen , G. Qian , X. Zhuo , H. Yang , S. Yin , Chem. Eng. J. 2022, 449, 137791.

[21] Y. Zhou , Y. Wang , D. Kong , Q. Zhao , L. Zhao , J. Zhang , X. Chen , Y. Li , Y. Xu , C. Meng , Adv. Funct. Mater. 2023, 33, 2210656.

[22] J. Wang , Y. Sun , Y. Qi , C. Wang , ACS Appl. Mater. Interfaces 2021, 13, 57392. 34806865 10.1021/acsami.1c18593

[23] M. Bollu , D. T. Tran , S. Prabhakaran , D. H. Kim , N. H. Kim , J. H. Lee , Nano Energy 2024, 123, 109413.

[24] F. Luo , S. Pan , Y. Xie , C. Li , Y. Yu , Z. Yang , J. Energy Chem. 2024, 90, 1.

[25] B. Zhu , Z. Liang , R. Zou , Small 2020, 16, 1906133. 10.1002/smll.201906133 31913584

[26] F. O. Boakye , M. G. Sendeku , A. Kumar , S. Ajmal , K. A. Owusu , K. B. Ibrahim , M. Tabish , F. uz Zaman , M. A. Mushtaq , K. M. Alotaibi , Appl. Catal. B: Environ. Energy 2024, 352, 124013.

[27] Y. Vlamidis , S. Forti , A. Rossi , C. Marinelli , C. Coletti , S. Heun , S. Veronesi , Part. Part. Syst. Charact. 2024, 41, 2400141.

[28] X. Zhang , Y. Ding , G. Wu , X. Du , Int. J. Hydrogen Energy 2020, 45, 30611.

[29] Q. Gan , X. Cheng , J. Chen , D. Wang , B. Wang , J. Tian , T. T. Isimjan , X. Yang , Electrochim. Acta 2019, 301, 47.

[30] K. B. Ibrahim , T. A. Shifa , M. Bordin , E. Moretti , H.‐L. Wu , A. Vomiero , Small Methods 2023, 7, 2300348. 10.1002/smtd.202300348 37350490

[31] F. Li , J. Chen , D. Zhang , W.‐F. Fu , Y. Chen , Z. Wen , X.‐J. Lv , Chem. Commun. 2018, 54, 5181. 10.1039/c8cc01404c 29610799

[32] D. Xu , Z. Fu , D. Wang , Y. Lin , Y. Sun , D. Meng , T. feng Xie , Phys. Chem. Chem. Phys. 2015, 17, 23924. 26309038 10.1039/c5cp03310a

[33] H. Bandal , K. K. Reddy , A. Chaugule , H. Kim , J. Power Sources 2018, 395, 106.

[34] K. Belay Ibrahim , T. Ahmed Shifa , S. Zorzi , M. Getaye Sendeku , E. Moretti , A. Vomiero , Prog. Mater. Sci. 2024, 144, 101287.

[35] X. Wang , Z. Li , S. Sun , H. Sun , C. Yang , Z. Cai , H. Zhang , M. Yue , M. Zhang , H. Wang , J. Colloid Interface Sci. 2024, 662, 596. 38367577 10.1016/j.jcis.2024.02.043

[36] W. Tang , J. Zou , Z. Li , X. Zhang , T. Xie , J. Li , X. He , X. Tang , X. Liu , W. Chu , Catal. Sci. Technol. 2024, 14, 2717.

[37] K. B. Ibrahim , T. A. Shifa , P. Moras , E. Moretti , A. Vomiero , Small 2023, 19, 2204765. 10.1002/smll.202204765 36354170

[38] S. Zou , M. S. Burke , M. G. Kast , J. Fan , N. Danilovic , S. W. Boettcher , Chem. Mater. 2015, 27, 8011.

[39] Y. Gan , Y. Ye , X. Dai , X. Yin , Y. Cao , R. Cai , B. Feng , Q. Wang , X. Zhang , Small 2023, 19, 2303250. 10.1002/smll.202303250 37464564

[40] L. Guo , J. Chi , J. Zhu , T. Cui , J. Lai , L. Wang , Appl. Catal., B 2023, 320, 121977.

[41] M. G. Sendeku , T. A. Shifa , F. T. Dajan , K. B. Ibrahim , B. Wu , Y. Yang , E. Moretti , A. Vomiero , F. Wang , Adv. Mater. 2024, 36, 2308101. 10.1002/adma.202308101 38341618

[42] Y. Tong , P. Chen , M. Zhang , T. Zhou , L. Zhang , W. Chu , C. Wu , Y. Xie , ACS Catal. 2018, 8, 1.

[43] X. Gao , X. Bai , P. Wang , Y. Jiao , K. Davey , Y. Zheng , S.‐Z. Qiao , Nat. Commun. 2023, 14, 5842. 37730706 10.1038/s41467-023-41588-w PMC10511637

[44] A. J. Hensley , K. Ghale , C. Rieg , T. Dang , E. Anderst , F. Studt , C. T. Campbell , J.‐S. McEwen , Y. Xu , J. Phys. Chem. C 2017, 121, 4937.

[45] L. Li , Q. Sun , X. Liu , C. Wu , H. Zhao , C. Zhang , Algorith. Architect. Parallel Proces. 2018, 18, 15.

[46] G. Kresse , D. Joubert , Phys. Rev. B 1999, 59, 1758.

[47] P. E. Blöchl , Phys. Rev. B 1994, 50, 17953. 10.1103/physrevb.50.17953 9976227

[48] K. Harrath , Z. Yao , Y.‐F. Jiang , Y.‐G. Wang , J. Li , J. Phys. Chem. C 2024, 128, 5579.

[49] K. Harrath , Z. Yao , Y.‐F. Jiang , Y.‐G. Wang , J. Li , J. Phys. Chem. Lett. 2023, 14, 4033. 37093648 10.1021/acs.jpclett.3c00375

[50] L. Bahri , F. Mbarki , K. Harrath , Chem. Pap. 2023, 77, 3759.

[51] S. Grimme , Wiley Interdiscip. Rev.: Comput. Mol. Sci. 2011, 1, 211.

[52] I. C. Man , H. Y. Su , F. Calle‐Vallejo , H. A. Hansen , J. I. Martínez , N. G. Inoglu , J. Kitchin , T. F. Jaramillo , J. K. Nørskov , J. Rossmeisl , ChemCatChem 2011, 3, 1159.

[53] M.‐C. Tsai , T.‐T. Nguyen , N. G. Akalework , C.‐J. Pan , J. Rick , Y.‐F. Liao , W.‐N. Su , B.‐J. Hwang , ACS Catal. 2016, 6, 6551.

[54] Y. Liu , Y. Ying , L. Fei , Y. Liu , Q. Hu , G. Zhang , S. Y. Pang , W. Lu , C. L. Mak , X. Luo , L. Zhou , M. Wei , H. Huang , J. Am. Chem. Soc. 2019, 141, 8136. 31017412 10.1021/jacs.8b13701

[55] H. Ren , Y. Pan , C. C. Sorrell , H. Du , J. Mater. Chem. A 2020, 8, 3154.

