# Supplementary material for: Ru‐Doped Fe₂TiO₅ as a High‐Performance Electrocatalyst for Urea‐Assisted Water Splitting
Source: Small. 2025 Mar 10;21(41):2412370. doi: 10.1002/smll.202412370 (PMC12530029; doi:10.1002/smll.202412370)
Supplement: Supplementary file 1 — Supporting Information [file SMLL-21-2412370-s001.docx]

**Supporting Information**

**Ru-Doped Fe₂TiO₅ as a High-Performance Electrocatalyst for Urea-Assisted Water Splitting**

### *Kassa Belay Ibrahim^a*^ Mohammadhossein Hamrang ^a^,* [*Karim Harrath*](https://www.nature.com/articles/s41467-024-49510-8#auth-Karim-Harrath-Aff4)*^b^, Matteo Bordin^a^, Tofik Ahmed Shifa*^a^* *,* [*Stéphanie Bruyère*](https://pubs.rsc.org/en/results?searchtext=Author%3ASt%C3%A9phanie%20Bruy%C3%A8re)*^c^, David Horwat^c^, Enrique Rodríguez-Castellón^d^, Marshet Getaye Sendeku^e^, Pratik Shinde^a^, Danilo Oliveira de Souza^g^,* *Luca Olivi^g^, Elisa Moretti^a*^, Alberto Vomiero^a,f*^*

^a^ Department of Molecular Sciences and Nanosystems, Ca’ Foscari University of Venice, Via Torino 155, 30170 Venezia Mestre, Italy

^b^ GanJiang Innovation Academy, Chinese Academy of Science, PR China

^c^ Université de Lorraine, CNRS, IJL, F-54000 Nancy, France

^d^ Departamento de Química Inorgánica, Cristalografia y Mineralogía, Facultad de Ciencias, Instituto Interuniversitario de Investigación en Biorrefinerías I3B, Universidad de Málaga, 29071 Málaga, Spain

^e^ Ocean Hydrogen Energy R&D Center, Research Institute of Tsinghua University in Shenzhen, Shenzhen, 518057, P. R. China

^f^ Division of Materials Science, Department of Engineering Sciences and Mathematics, Luleå University of Technology, SE-97187 Luleå, Sweden

^g^ Elettra-Sincrotrone Trieste, Strada Statale 14, km 163.5, 34149, Trieste, ITALY

**
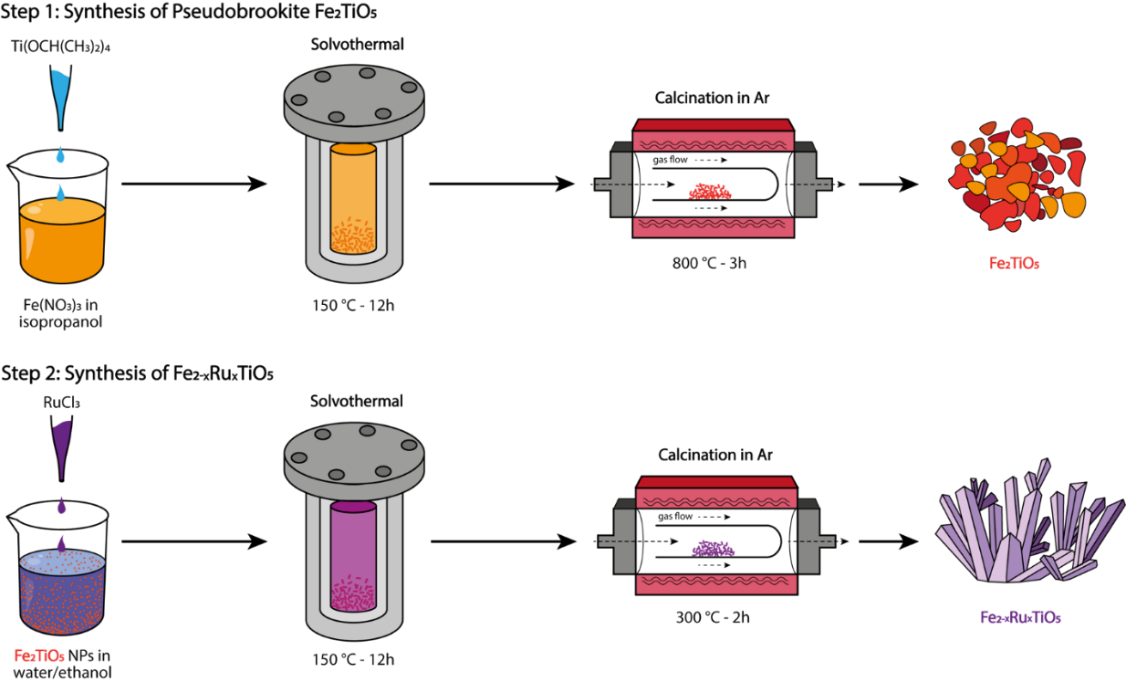
**

Scheme S1**.** The synthesis process of Fe_2-x_Ru_x_TiO_5_ via hydrothermal method accompanied by CVD treatment.

**
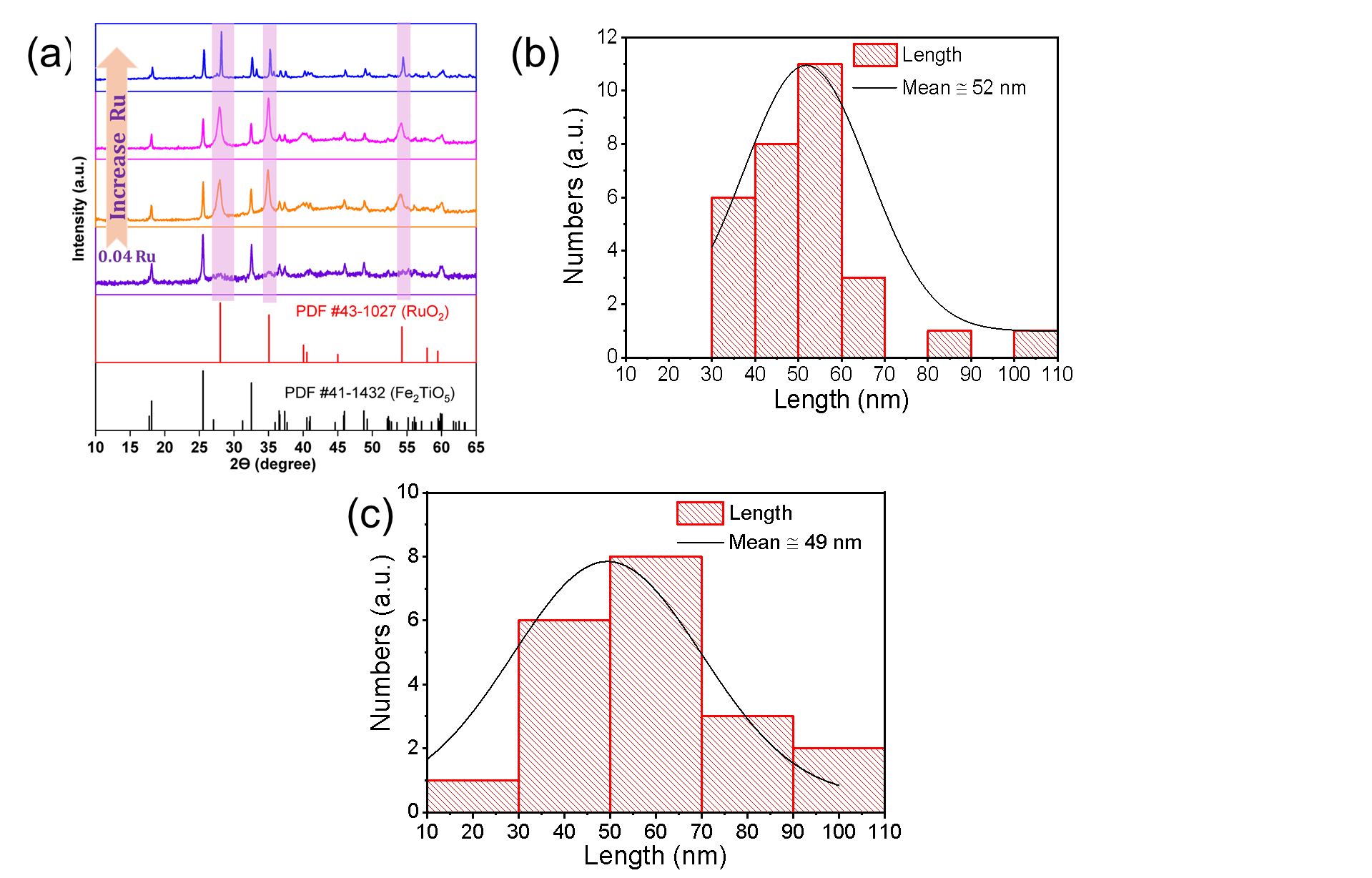
**

Figure S1. (a) XRD with increasing Ru content. Particle size distribution (b). Fe_2_TiO_5_ (c). Fe_2-x_Ru_x_TiO_5_


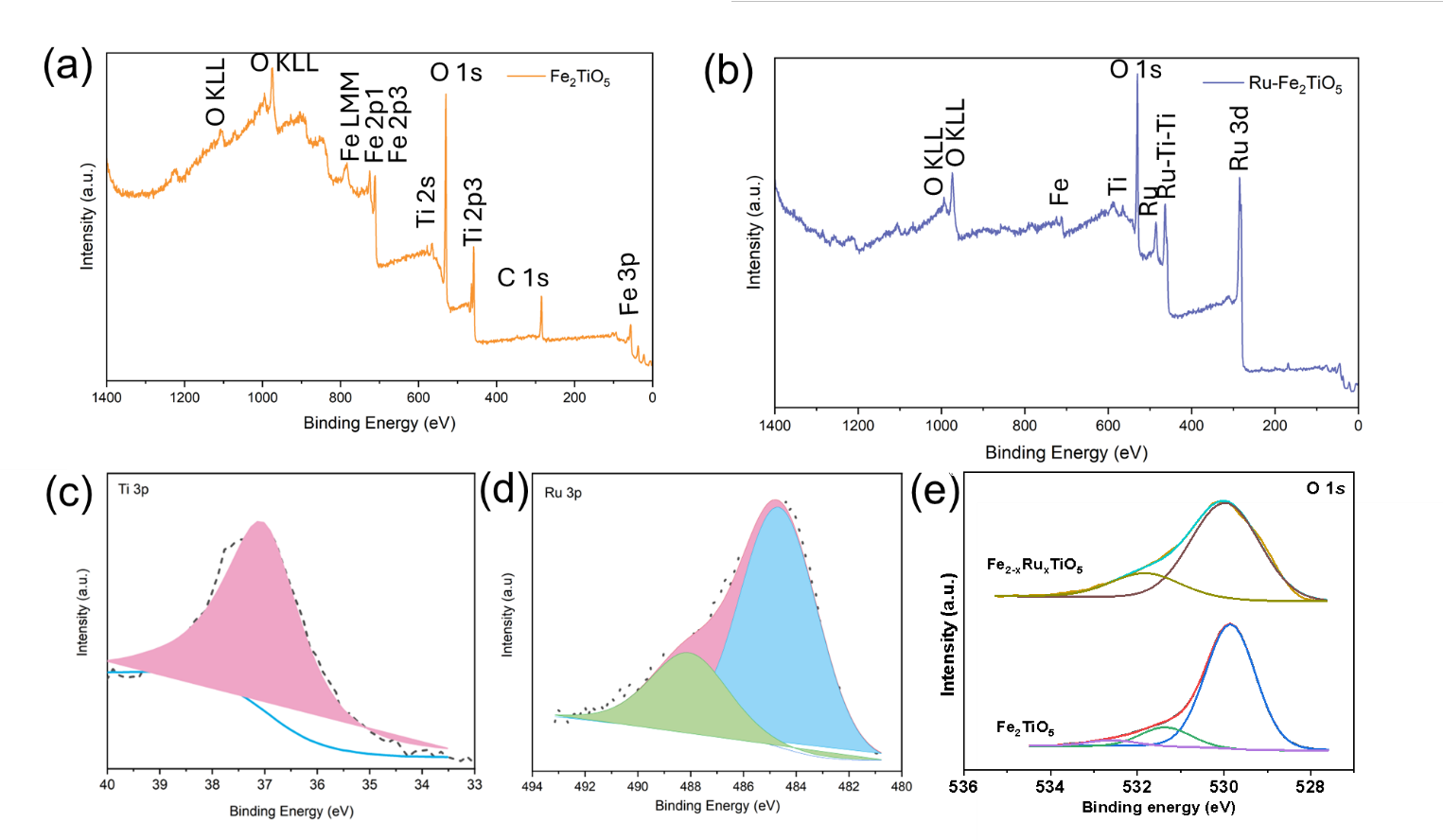


Figure S2. XPS wide range spectra (a) Fe_2_TiO_5_ (b). Fe_2-x_Ru_x_TiO_5_ (c). O 1s (d). Ti 3*p* spectra (e). Ru 3*p* spectra.


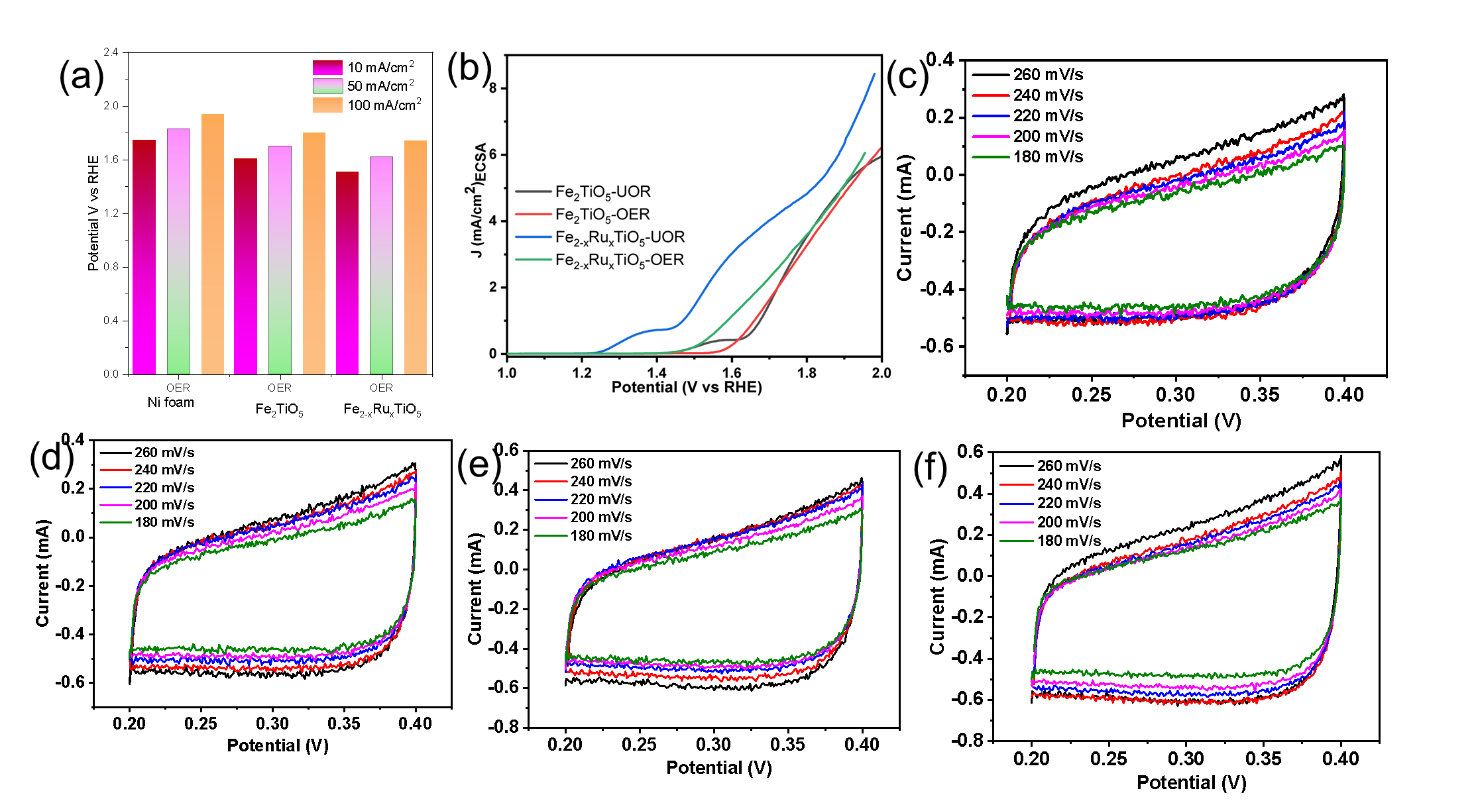


Figure S3. (a) Comparison of UER potentials at 10, 50, and 100 mA cm^−2^ current densities. (b) LSV normalized by ECSA. Cyclic voltammetry (CV) curves under UOR (c) Fe_2_TiO_5_ (d) Fe_2-x_Ru_x_TiO_5_. Under OER (e) Fe_2_TiO_5_ (f) Fe_2-x_Ru_x_TiO_5_ was collected at various scan rates (mV s^-1^).


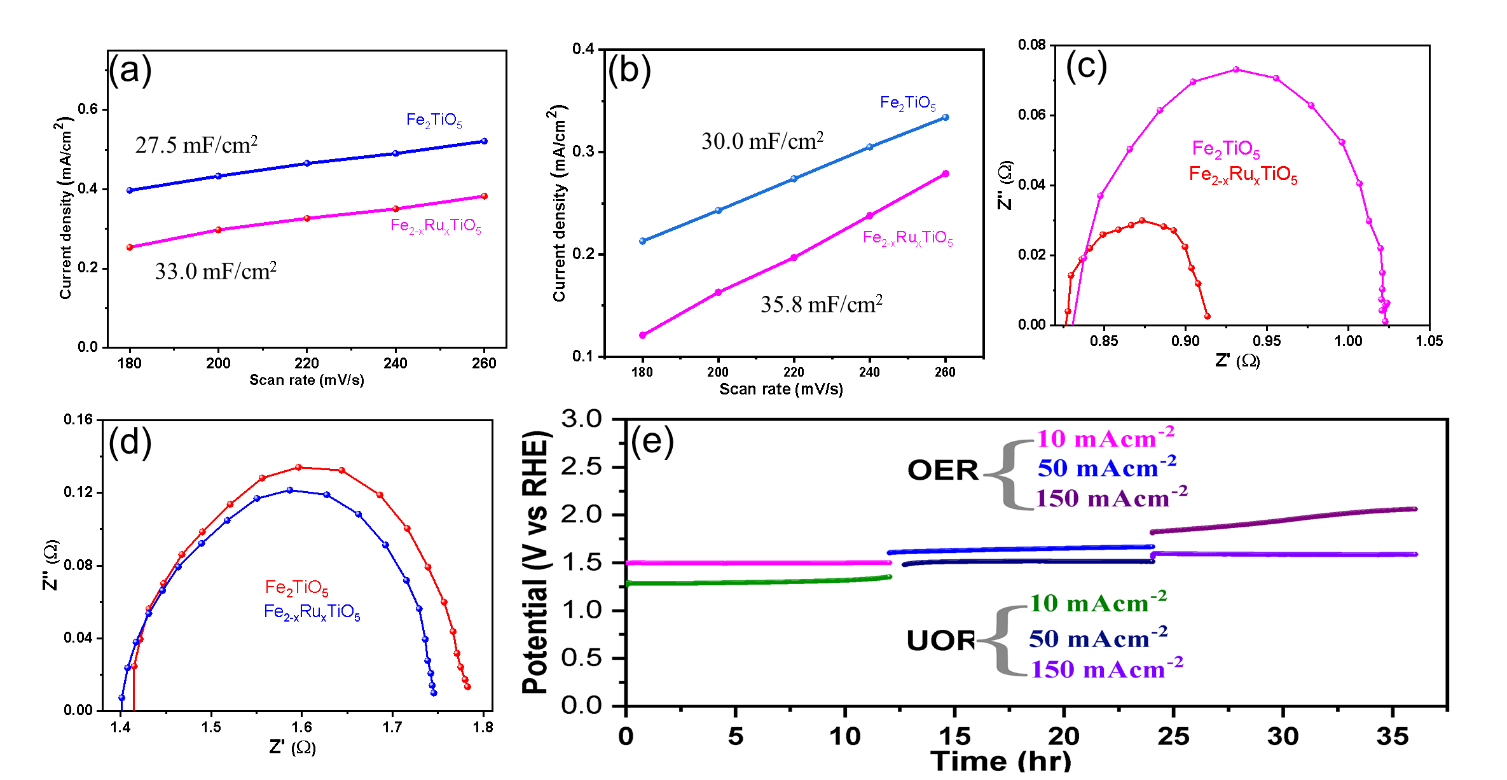


Figure S4. Double-layer capacitance (C_dl_) measurements of (a) UOR (b) OER. Comparison of Nyquist plots for (c) UOR (d). OER (e) Chronopotentiometric run of Fe_2-x_Ru_x_TiO_5_ for the long-term durability test at 10, 50, and 100 mA cm*^−^*^2^.


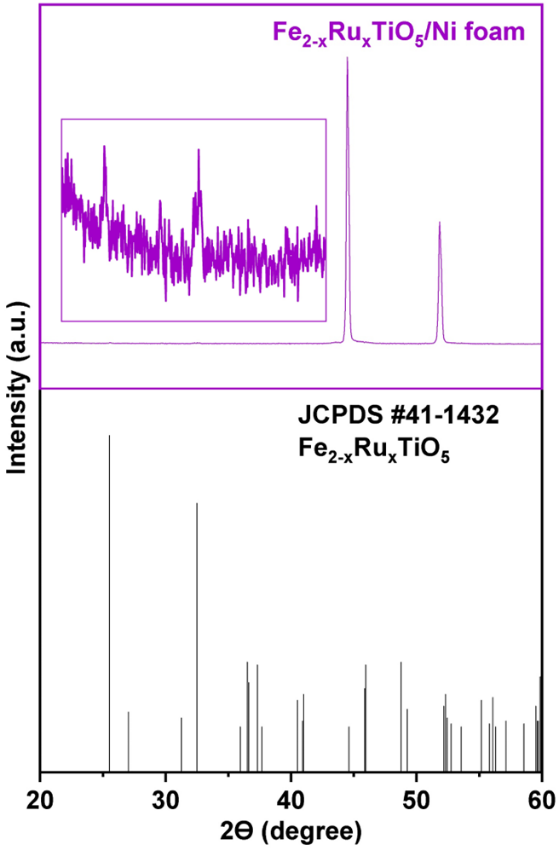


Figure S5. post UOR XRD pattern for Fe_2-x_Ru_x_TiO_5_

**TABLE S1:** Comparison of UOR/OER Stability with various recently reported literatures.

| R/No. | Materials | Stability (hr) | | Ref. |
| --- | --- | --- | --- | --- |
|  |  | 1M KOH (OER) | 1 M KOM +0.5 M Urea (UOR) |  |
| 1 | Mo-NiS/CF | 24 h @100 mA cm^−2^ | | ^1^ |
| 2 | CoMn/CoMn_2_O_4_ | 16 h @20 mA cm^−2^ | | ^2^ |
| 3 | Ni-SO_X_ | 80 h@50 mA cm ^−2^ | | ^3^ |
| 4 | MoNi_4_ | 40 h@10 mA cm^−2^ | | ^4^ |
| 5 | Ni_x_Co_2-x_P/C | 6 h@10 mA cm^−2^ | | ^5^ |
| 6 | S-MnO_2_-G-NF | 16 h @1.4 V | | ^6^ |
| 7 | SeFe-Ni_3_S_2_ | 40 h@1.47 V | | ^7^ |
|  |  | 60h @1.365 V | |  |
| 8 | Hf-NiFe-LDH/NF | 90 h@100mA/cm^2^ | | ^8^ |
|  |  | 7.5h@10 mA/cm^2^ | |  |
| 9 | NiO-SnO_2_ | 20 h@10 mA/cm^2^ | | ^9^ |
| 10 | Ni_0.75_Fe_0.25_Se_2_/CC | 48 h@1.4 V | | ^10^ |
| 11 | Fe_2-x_Ru_x_TiO_5_ | 72hr 1.5 V | | **This work** |
|  |  | 36 hr @ 10, 50, 150 mA/cm^2^ | |  |

Reference

1. Zhou, Y.; Wang, Y.; Kong, D.; Zhao, Q.; Zhao, L.; Zhang, J.; Chen, X.; Li, Y.; Xu, Y.; Meng, C., *Advanced Functional Materials* **2023,** 33 (8), 2210656.

2. Wang, C.; Lu, H.; Mao, Z.; Yan, C.; Shen, G.; Wang, X., *Advanced Functional Materials* **2020,** 30 (21), 2000556.

3. Gao, X.; Bai, X.; Wang, P.; Jiao, Y.; Davey, K.; Zheng, Y.; Qiao, S.-Z., *Nature Communications* **2023,** 14 (1), 5842.

4. Wang, X.; Sha, N.; Zhao, N.; Su, T.; Lv, C.; Yang, L.; Xie, Y.; Ye, K., *Chemical Engineering Journal* **2024,** 499, 156119.

5. Rezaee, S.; Shahrokhian, S., *Nanoscale* **2020,** 12 (30), 16123-16135.

6. Chen, S.; Duan, J.; Vasileff, A.; Qiao, S. Z., *Angewandte Chemie International Edition* **2016,** 55 (11), 3804-3808.

7. Xu, S.; Jiao, D.; Ruan, X.; Jin, Z.; Qiu, Y.; Fan, J.; Zhang, L.; Zheng, W.; Cui, X., *Journal of Colloid and Interface Science* **2024,** 671, 46-55.

8. Wang, J.; Wang, M.; Lu, Z.; Xie, J.; Huang, J.; Hu, J.; Cao, Y., *International Journal of Hydrogen Energy* **2024,** 82, 724-732.

9. Naresh, B.; Sreekanth, T.; Suma, C.; Kumar, K. S.; Yoo, K.; Kim, J., *Journal of Alloys and Compounds* **2025,** 1010, 177865.

10. Zhu, M.; Tang, P.; Li, X.; Zhao, M.; Liu, L.; Tang, W.; Xu, Z.; Dong, G.; Yang, M.; Shi, Z., *International Journal of Hydrogen Energy* **2025,** 102, 626-634.
